# High-efficiency dual-modes vortex beam generator with polarization-dependent transmission and reflection properties

**DOI:** 10.1038/s41598-018-24929-4

**Published:** 2018-04-23

**Authors:** Shiwei Tang, Tong Cai, Guang-Ming Wang, Jian-Gang Liang, Xike Li, Jiancheng Yu

**Affiliations:** 1 Department of Physics, Faculty of Science, Ningbo University, Ningbo, 315211 China; 2 Air and Missile Defend College, Air force Engineering University, Xi’ an, 710051 China

## Abstract

Vortex beam is believed to be an effective way to extend communication capacity, but available efforts suffer from the issues of complex configurations, fixed operation mode as well as low efficiency. Here, we propose a general strategy to design dual-modes vortex beam generator by using metasurfaces with polarization-dependent transmission and reflection properties. Combining the focusing and vortex functionalities, we design/fabricate a type of compact dual-modes vortex beam generator operating at both reflection/transmission sides of the system. Experimental results demonstrate that the designed metadevice can switch freely and independently between the reflective vortex with topological charge *m*_1_ = 2 and transmissive vortex with *m*_2_ = 1. Moreover, the metadevice exhibits very high efficiencies of 91% and 85% for the reflective and transmissive case respectively. Our findings open a door for multifunctional metadevices with high performances, which indicate wide applications in modern integration-optics and wireless communication systems.

## Introduction

Orbital angular momentum (OAM) vortex beam is very essential in modern science and technology, since it is an effective way to extend the communication capacity without increasing the bandwidth^1–3^. A prominent attribute of a vortex beam is a phase singularity indicating a dark core with zero intensity along the beam-axis together with an annular transverse intensity profile^4^. The unique property of vortex beam has led to many fascinating applications, such as plasmon excitation^5^, improved focusing^6^, information processing^7–9^, and radio frequency communication^10,11^. To date, vortex beams has been generated by spiral phase plates^12,13^, holograms^14^, spiral reflectors as well as antenna arrays^15,16^. However, these devices suffer from complex configurations, large structure size, single functionality and low efficiency. Therefore, a scientific issue is that, how to realize a simple and compact vortex beam generator with multifunction and high efficiency?

Metasurfaces, as a planar version of metamaterials, have provided strong capabilities to manipulate the wavefronts of electromagnetic (EM) waves through their desirable control on the local phases^17–30^. Very recently, metasurfaces have been found widely applicated in designing multifunctional metadevices working at frequencies ranging from microwave to visible^31–38^, which can be dynamically tuned by external condition^39–43^. Diversified functionalities (i.e. beam bending and focusing) are predesigned on a single device with subwavelength thickness based on the polarization-dependent metasurface^32^. Such great wave-front manipulation capability of metasurfaces potentially provides an alternative to construct vortex beam generator with multifunctionalities. In this regard, multiple vortex beams are constructed by supervising the phase functions of different topological modes^1,3^. However, the vortex beams can only be manipulated either at reflection side or transmission side of the metasurface, leaving the other space uncontrolled. Moreover, the simple superposition of phase profiles decreases the working efficiencies of these devices.

**Figure 1:**
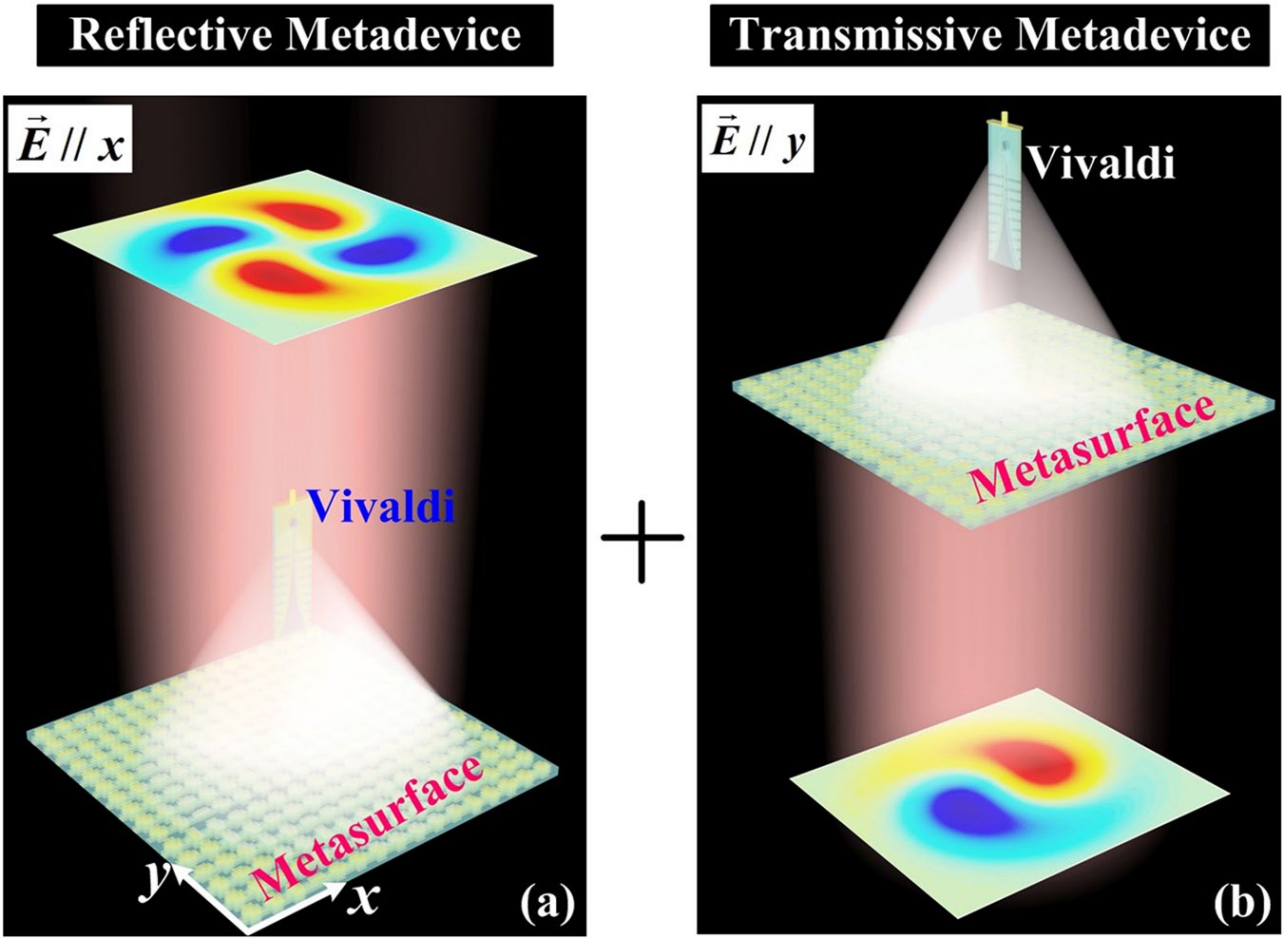
Schematics and working principle of the dual-modes vortex beam generator. Our vortex beam generator consists of Vivaldi antenna (feed antenna) and bifunctional metasurface. (**a**) Under E//x excitation, our device can work at reflection geometry, behaving as a reflective vortex beam generator with m_1_ = 2. (**b**) Under E//y excitation, our device can work at transmission geometry, behaving as a transmissive vortex beam generator with m_2_ = 1.

In this work, we propose a new strategy to design high-performance dual-modes vortex beam generator based on the metasurface which can manipulate the transmitted and reflected EM waves independently depending on the incident polarizations^33^. For practical applications, we fixed a focusing phase-distribution and vortex phase-distribution with different topological charges (denoted by *m*) along two orthogonal polarizations at a single metasurface, respectively. As shown in Fig. 1(a), shining an *x*-polarization quasi-spherical wave normally onto our metasurface, a reflective vortex beam generator with topological charge *m*_1_ = 2 can be obtained. Meanwhile, for a *y*-polarized incident wave, our metadevice can work as a transmissive vortex beam generator with *m*_2_ = 1 (see Fig. 1(b)). Moreover, our metadevice can work with very high efficiencies since our meta-atom is totally reflective for *x*-polarization incident wave and completely transparent for *y*-polarization EM wave with arbitrary phase profiles, respectively. Our findings can not only set up a new platform to design compact and integrated vortex beam generator, but more importantly, they also provide a powerful guideline to design other metadevices with other functionalities or in other frequency domains.

## Working principle and meta-atom design

In order to realize the above mentioned dual-modes vortex beam generator, we need to design an appropriate meta-atom which can control the transmitted and reflected waves efficiently and independently. In our recent research, we proposed such a meta-atom, which can meet the above requirements^33^. Here, we describe its working principle briefly. For a system with mirror symmetry, its EM property can be described by two diagonal Jones matrices $$R=(\begin{array}{cc}{r}_{xx} & 0\\ 0 & {r}_{yy}\end{array})$$ and $$T=(\begin{array}{cc}{t}_{xx} & 0\\ 0 & {t}_{yy}\end{array})$$, with *r*_*xx*_, *r*_*yy*_, *t*_*xx*_ and *t*_*yy*_ being the reflection and transmission coefficients with polarizations along two principle axes $\hat{x}$ and $\hat{y}$, respectively. In a lossless system, we can obtain $|{r}_{xx}{|}^{2}+|{t}_{xx}{|}^{2}=1$ and $|{r}_{yy}{|}^{2}+|{t}_{yy}{|}^{2}=1$ due to the energy conservation. Here, we consider two ideal conditions. Firstly, for an *x*-polarized incident wave, we can completely suppress the transmission power $(|{t}_{xx}|=0)$ to achieve pure reflection $(|{r}_{xx}|=1)$. Secondly, for a *y*-polarization, we can enlarge the transmission part $(|{t}_{yy}|=1)$ and block all the reflection $(|{r}_{yy}|=0)$. These two conditions are independent and can be combined to manipulate the transmitted and reflected waves simultaneously.

Then we discuss how to design such a desirable meta-atom. Here, meta-atoms in multilayer geometry (with deep-subwavelength total thicknesses) are found as alternative candidates. Each single layer exhibits perfect EM transmission at a particular frequency due to the interaction between the patch resonator and the opaque mesh. Mutual interactions between different layers can realize high transmission amplitude as well as the large transmission-phase variation range covering 360°.

**Figure 2:**
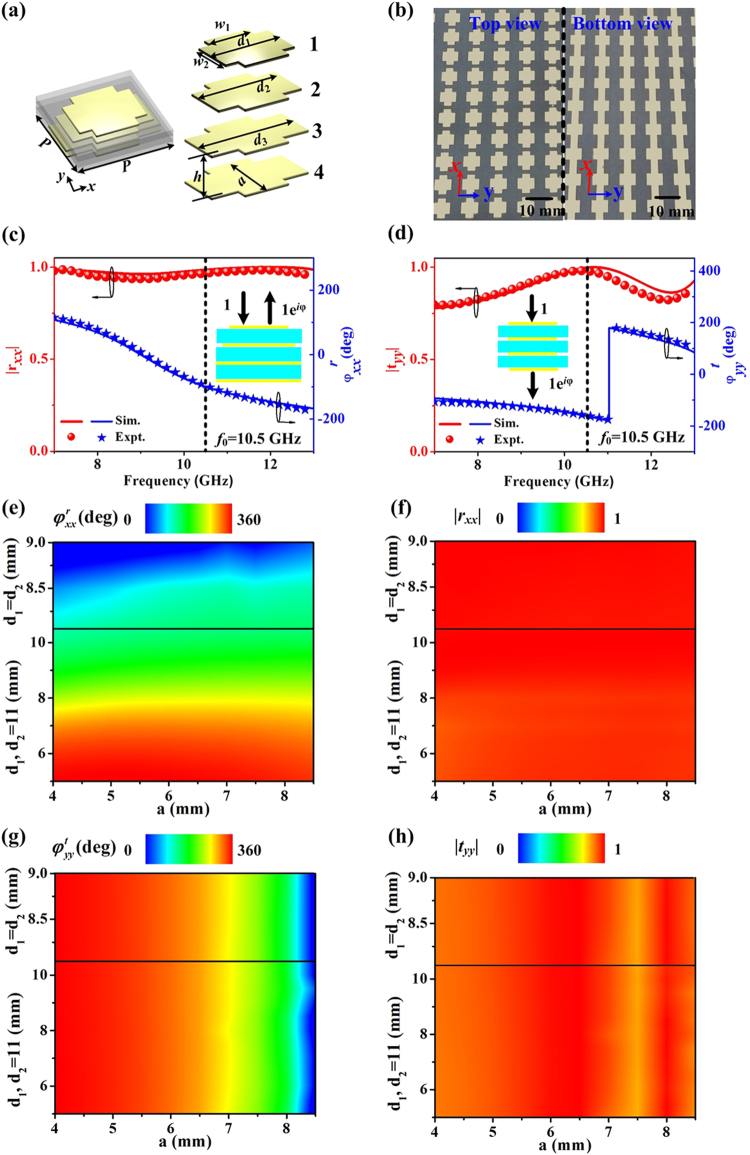
Design and characterization of the proposed meta-atom. (**a**) Schematics of the proposed meta-atom composed by four metallic layers separated by three F4B spacers. The following geometrical parameters are fixed as: *w*_1_ = 5 mm, *w*_2_ = 4 mm, *d*_3_ = *d*_4_ = *P* = 11 mm. Other parameters (*d*_1_,*d*_2_ and lengths of all y-orientated bars *a*) are tuned appropriately in designing each meta-atom. (**b**) Top view (left) and bottom view (right) pictures of the fabricated metasurface consisting of a periodic array of meta-atoms with *a* = 6.3 mm, *d*_1_ = 9 mm and *d*_2_ = 11 mm. Measured and FDTD simulated amplitude/phase spectra of (**c**) reflection and (**d**) transmission for metasurface under excitations with different polarizations. FDTD simulated maps of (**e**) reflection phase ${\phi }_{xx}^{r}$, (**f**) reflection amplitude $|{r}_{xx}|$, (**g**) transmission phase ${\phi }_{yy}^{t}$ and (**h**) transmission amplitude $|{t}_{yy}|$ by interchanging *d*_1_ and *d*_2_ under different polarizations at 10.5 GHz.

Figure 2(a) depicts the schematic of the proposed meta-atom. The meta-atom consists of four metallic layers which are separated by three 1.5 mm-thick F4B dielectric substrates $({\varepsilon }_{r}=2.65+0.01i)$. At *x* direction, we connect the metallic stripes at bottom two layers to block the *x*-polarized incident wave, while the metallic stripes at upper two layers have small size to tune the reflected wave. In our design, the parameter *d*_1_ and *d*_2_ can be tuned to control the number of magnetic resonances and expand the design freedom. At *y* direction, the metallic patch structures have the same size, which can enhance the transmission and enlarge the phase-shift range due to the coupling among different layers^44–48^. To demonstrate our concept, we fabricate a microwave sample, which consists of periodic array of such special meta-atoms, with its top and bottom views shown in Fig. 2(b). The measured reflection coefficient under an *x*-polarized EM wave is plotted in Fig. 2(c). We can see that the reflection amplitude $|{r}_{xx}|$ reaches near 1 at the target frequency of *f*_0_ = 10.5 GHz, indicating high reflection for our meta-atom. The reflection phase ${\phi }_{xx}^{r}$ varies from −180° to 180° as the frequency changes from 7 GHz to 13 GHz. Under a *y*-polarized incident wave, our metasurface can transmit nearly all signal $(|{t}_{yy}|=0.95)$ at *f*_0_ = 10.5 GHz, which is demonstrated by measured transmission amplitude shown in Fig. 2(d). Moreover, the transmission phase ${\phi }_{yy}^{t}$ covers 360° range as frequency varies. Finite-difference-time-domain (FDTD) simulation results agree well with the measured cases.

After characterizing the EM response of this typical meta-atom, we can immediately understand that the phases $({\phi }_{xx}^{r}\,{\rm{and}}\,{\phi }_{yy}^{t})$ can be tuned by changing the geometric structural details. Figure 2(e–h) illustrate, respectively, how ${\phi }_{xx}^{r}$, $|{r}_{xx}|$, ${\phi }_{yy}^{t}$, and $|{t}_{yy}|$ vary against the parameters *a*, *d*_1_ and *d*_2_, with frequency fixed at 10.5 GHz. Obviously, ${\phi }_{xx}^{r}$ is sensitive to *d*_1_ and *d*_2_ but insensitive to *a*, while ${\phi }_{yy}^{t}$ behaves oppositely. At the top of Fig. 2(e–h), reflection response of the designed metasurface is determined by two magnetic resonances which is generated by varying *d*_1_ and *d*_2_ simultaneously (*d*_1_ = *d*_2_). While at the bottom of Fig. 2(e–h), it is determined by the single magnetic resonance generated by changing the parameter *d*_1_ and fixing *d*_2_ = 11 mm. Changing the structural parameters within the restricted spaces (5 mm < *d*_1_ < 10.8 mm, *d*_2_ = 11 mm and 8.2 mm < *d*_1_ = *d*_2_ < 9 mm and 4 mm < a < 8.5 mm), we find that the variations of the two phases $({\phi }_{xx}^{r}\,{\rm{and}}\,{\phi }_{yy}^{t})$ already cover the whole 360° range, while simultaneously the reflection/transmission amplitudes ($|{r}_{xx}|$ and $|{t}_{yy}|$) remain at very high values ($|{r}_{xx}|$ >0.92, $|{t}_{yy}|$ >0.85), which ensures the high working efficiency of the designed metasurface. With arbitrary phase distribution, coupled with high reflection/transmission amplitude of the meta-atom, we can achieve required functionalities as expected.

## Experimental Results and Discussions

**Figure 3:**
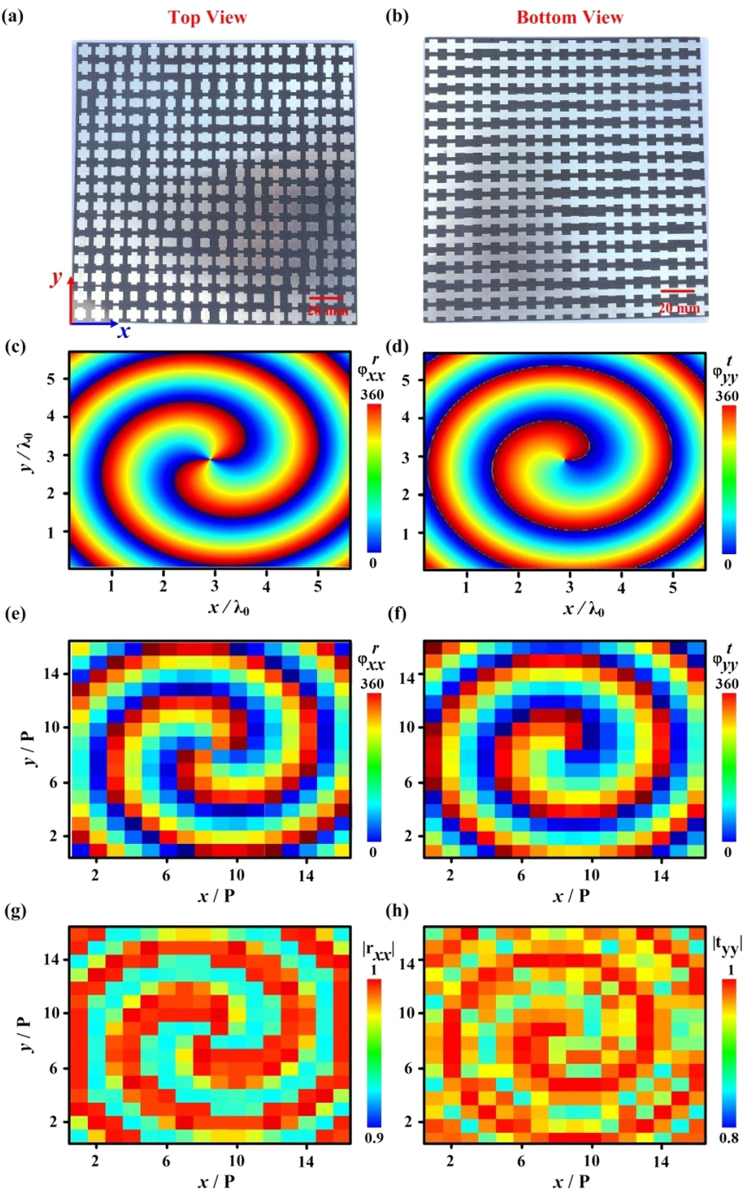
Design of dual-modes vortex beam generator. (**a**) Top-view and (**b**) bottom-view pictures of our designed/fabricated meta-device. (**c**,**d**) Continuous and (**e**,**f**) discrete phase distributions of (**c**,**e**) ${\phi }_{xx}^{r}$ and (**d**,**f**) ${\phi }_{yy}^{t}$ for the designed metasurface. The corresponding amplitude distributions of (**g**) |r_xx_| and (**h**) |t_yy_| at each meta-atom of metadevice.

The proposed meta-atom is very suitable to achieve bifunctional metadevices working at both reflection and transmission sides of the metasurfaces. Here, we construct a bifunctional vortex beam generator as an example. Different from currently reported vortex beam generator^1–16^, our design can not only realize vortex beams with different topological charges, but also with very high efficiencies, which results from the independent manipulation of vortex beams based on the polarization-dependence of the metasurfaces. To ensure a compact structure, we excite the metasurface with a self-made Vivaldi antenna, which can radiate quasi-spherical waves at a wide frequency range^49^. Thus, the metasurface should incorporate two distinct phase profiles of a lens and a vortex plate, which can be calculated as

1 $$\{\begin{array}{c}{\phi }_{xx}^{r}={k}_{0}(\sqrt{{{F}_{1}}^{2}+{y}^{2}+{x}^{2}}-{F}_{1})+{m}_{1}\cdot {\tan }^{-1}(y/x)\\ {\phi }_{yy}^{t}={k}_{0}(\sqrt{{{F}_{2}}^{2}+{y}^{2}+{x}^{2}}-{F}_{2})+{m}_{2}\cdot {\tan }^{-1}(y/x)\end{array}$$

with *F*_1_ and *F*_2_ being two focal lengths which can be chosen freely and arbitrarily, *m*_1_ and *m*_2_ denoting the topological charges which are integers. Here, we set *F*_1_ = *F*_2_ = 60 mm to ensure efficient integration of different functionalities. Moreover, *m*_1_ = 2 and *m*_2_ = 1 are chosen to demonstrate independent control of vortex modes. The designed metasurface consists of 16 × 16 meta-atoms and exhibits a total size of 176 × 176 mm^2^, with the pictures shown in Fig. 3(a) and (b). To validate our design, we show in Fig. 3(e,f) the FDTD calculated continuous phase distributions and discrete phase profiles at each meta-atom, which agree well with the theoretical values in Eq. (1). The corresponding high reflection-transmission amplitude distributions shown in Fig. 3(g,h) (|*r*_*xx*_| > 0.92, |*t*_*yy*_| > 0.86) indicate high working efficiencies of our metadevice.

**Figure 4:**
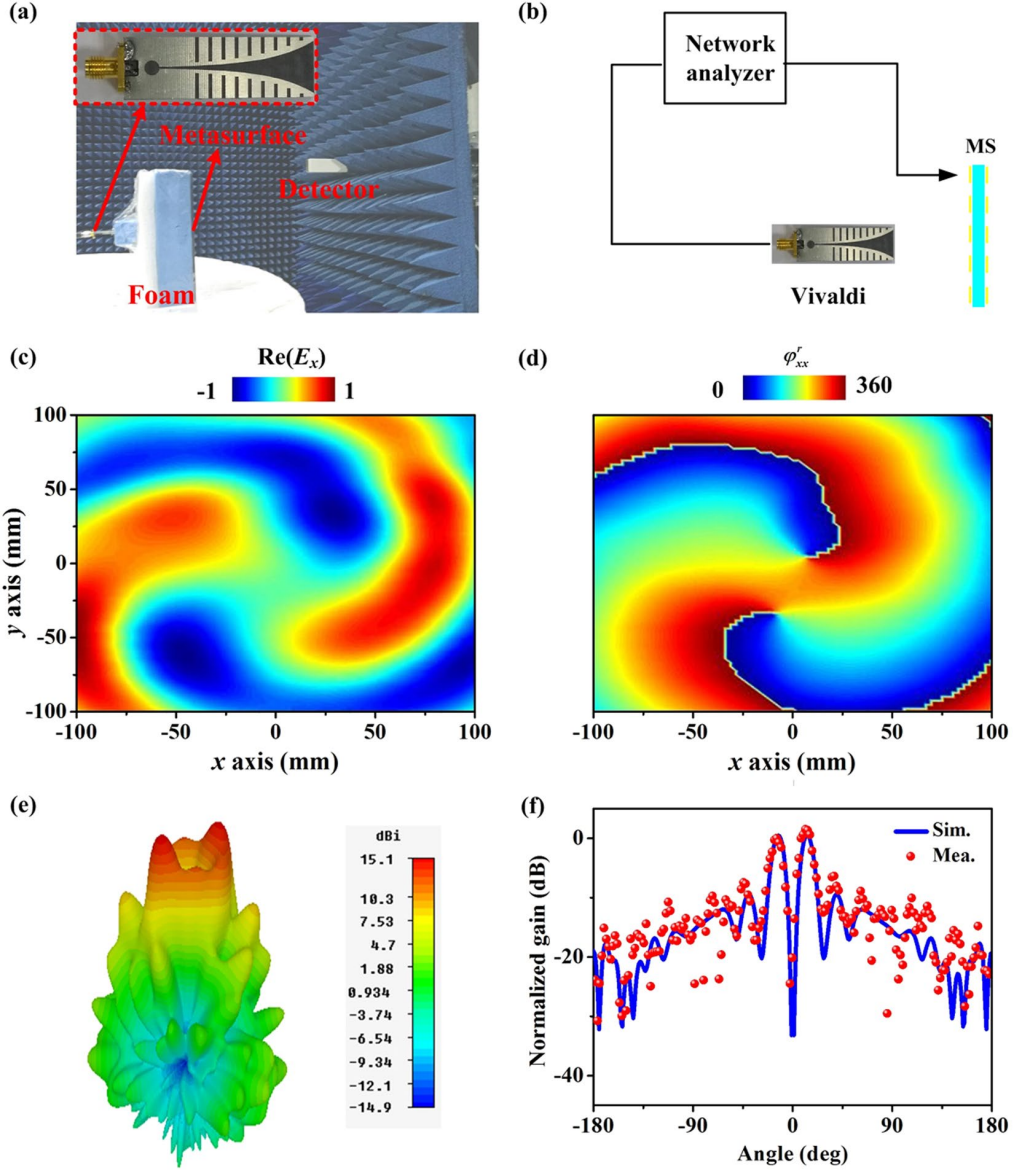
The performance of the reflection functionality of the designed metadevice. (**a**,**b**) Experimental setup of the vortex beam generator. Inset to (**a**) shows the picture of the fabricated feed antenna. Measured (**c**) Re(*Ex*) and (**d**) phase distributions on an xy-plane (30 cm behind the metasurface) at 10.5 GHz. (**e**) FDTD simulated 3-D radiation pattern of the metadevice under an x-polarized feed antenna. (**f**) Measured and simulated 2-D radiation patterns at xoz plane at 10.5 GHz.

With the fabricated sample in hand, the next step is to characterize the performances of the metadevices through microwave experiments. We firstly characterize its reflection functionality. As illustrated in Fig. 4(b), the designed vortex beam generator is excited by an *x*-polarized Vivaldi antenna. The electric field of the system is detected at the reflection side by a waveguide antenna and recorded by a vector-field network analyzer (Agilent E8362C PNA). Referring to the measured Re(*E*_*x*_) and phase distributions at *xoy* plane (30 cm behind the sample) shown in Fig. 4(c) and (d), we can see clearly that pure vortex beams with *m*_1_ = 2 are generated by integrating *x*-polarized waves reflected by meta-atoms at different positions. The amplitude null in the center of the 3-D radiation pattern in Fig. 4(e) demonstrates once again the excellent vortex effect for a second time. The measured 2-D radiation pattern at *xoz* plane agrees well with the simulated case, indicating that the amplitude in the azimuth (θ = 0°) is lower than −28 dB. Based on the far-field measurements, we can quantitatively examine the working efficiencies of our device. Here, there are three channels to propagate the energy of the incident waves: scattering to the transmission part, absorption and conversion to vortex beams by the reflection energy. For the reflective functionality, the working efficiency can be estimated by the formula η = 1 − T − A, where T and A describe the scattering energy to the transmission side and absorption, respectively. The transmission energy can be evaluated by integrating the energy at the transmission part of the far-field pattern, where T is found of 7% (5%) for measurement (simulation). The absorption is calculated as 2% (1.5%), estimated by integrating the total scattering energy with and without metasurfaces. Therefore, the numerical and experimental efficiency is estimated as 91% and 93.5%, respectively.

**Figure 5:**
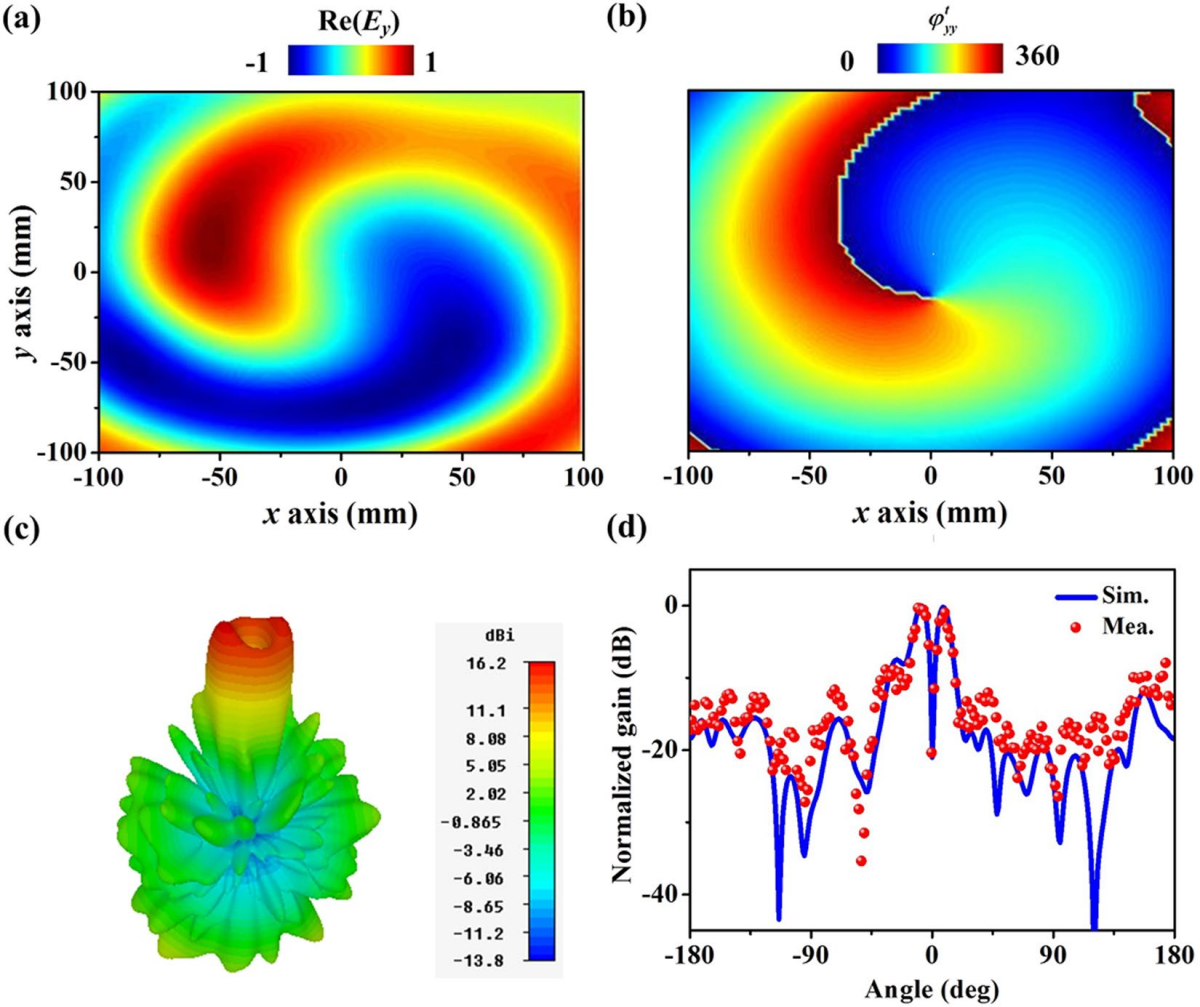
The performance of the transmission functionality of the designed metadevice. Measured (**a**) Re(*Ey*) and (**b**) phase distributions on an xy-plane (30 cm front of the metasurface) at 10.5 GHz. (**c**) FDTD simulated 3-D radiation pattern of the metadevice under a y-polarized feed antenna. (f) Measured and simulated 2-D radiation patterns at xoz plane at 10.5 GHz.

We secondly characterize the transmission property of our metadevice. Rotating the feeding Vivaldi antenna by 90°, our system can behave as a transmissive vortex beam generator with *m*_2_ = 1. Similar to the experimental configuration of the reflection functionality, we measure the electric field distributions at the transmission part (30 cm in front of the designed metadevice) (see Fig. 4(a)). The pure Re(*E*_*y*_) distribution in Fig. 5(a) and spiral trend of phase distribution in Fig. 5(b) demonstrate the excellent characteristic of vortex beam. Referring to the 3-D radiation pattern in Fig. 5(c), the deep dip at the center further validates the good performance of the designed system. Observation from the measured and simulated 2-D radiation patterns at *xoz* planes indicate that the gain is lower than −22 dB and −23 dB at the specular direction, respectively. The working efficiency can be described as η = 1 − R − A, with R denoting the reflection energy. R is computed by integrating the power at the reflection part of the designed system. Thus, the efficiency of the transmissive vortex beam is evaluated as 86% (89%) for measurement (simulation). Compared with the reported multi-modes vortex beam generators^1–16^, our design exhibits very high working efficiencies since the interferences among different modes are reduced based on the polarization-dependent property of the metadevice.

**Figure 6:**
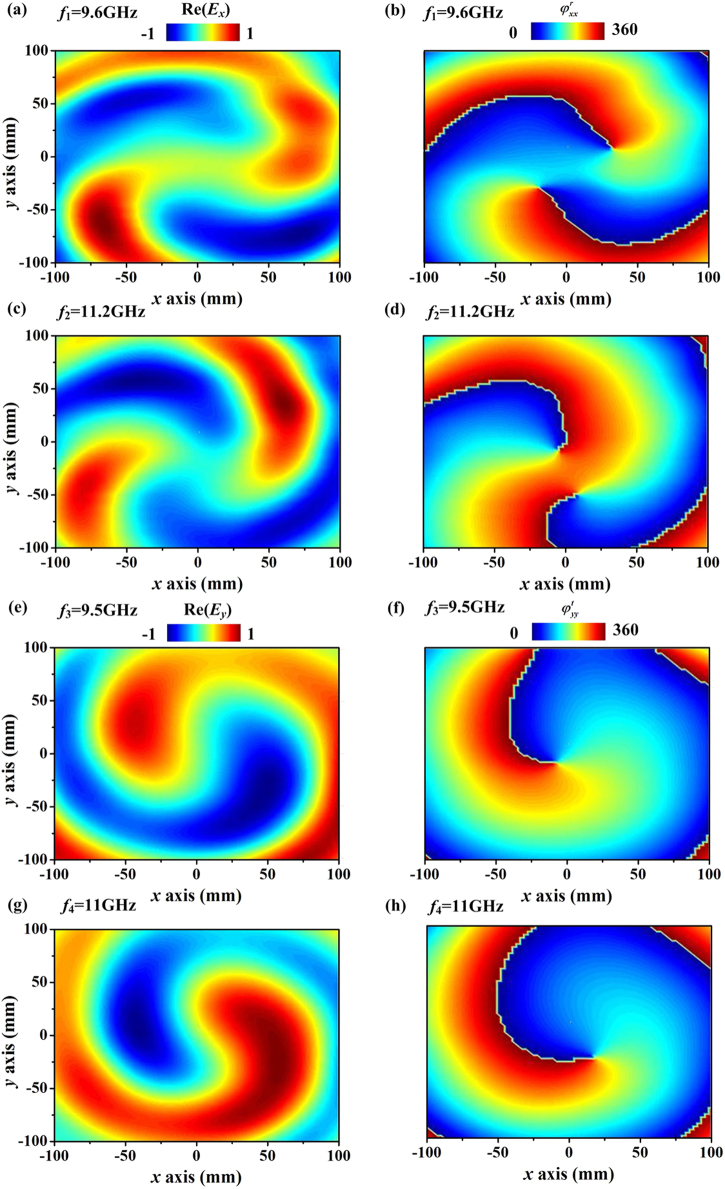
Evaluation of the bandwidth of the dual-modes vortex beam generator. Measured (**a**,**c**) Re(*Ex*) and (**b**,**d**) phase distributions on an xy-plane 30 cm behind of the metasurface at (**a**,**b**) 9.6 GHz and (**c**,**d**) 11.2 GHz under excitation of an x-polarized feed antenna. Measured (**e**,**g**) Re(*Ey*) and (**f**,**h**) phase distributions on an xy-plane 30 cm front of the metasurface at (**e**,**f**) 9.5 GHz and (**g**,**h**) 11 GHz under excitation of a y-polarized feed antenna.

Finally, we estimate the working bandwidth of our dual-modes vortex beam generator. The bandwidth is defined by −10 dB level of specular radiation for the far-field pattern. The bandwidth for the reflection functionality is found as 1.6 GHz (9.6–11.2 GHz). The corresponding Re(*E*_*x*_) and phase distributions at lower and higher frequencies of the band are shown in Fig. 6(a–d). Within the working frequency interval, the reflected waves can be successfully converted to vortex beams and keep at very high working efficiencies. Outside the working frequency range, specular reflections would increase significantly. The bandwidth of the transmission functionality varies from 9.5 GHz to 11 GHz. Similarly, the transmissive waves can be converted to pure vortex beams within the bandwidth, demonstrated by the pure Re(*E*_*y*_) and spiral phase distributions at two edges of frequency band shown in Fig. 6(e–h).

## Conclusion

To summarize, we proposed a new kind of dual-modes vortex beam generator by using well designed metasurfaces with polarization-dependent transmission and reflection properties. The designed vortex beam generator can not only manipulate the operating modes (topological charges) freely and independently but also can work at both sides of the metadevice. Both near-field and far-field characterizations demonstrate the good vortex effects. More importantly, the designed vortex beam generator exhibits very high working efficiencies (91% for the reflective mode and 85% for the transmissive case). Our findings pave the road to realize high-efficiency metadevices with mode-manipulation properties, which are crucial importance in modern EM integration.

### Data availability statement

All relevant data are within the paper.
